# Nutritional status of hospitalized elderly patients in Ethiopia: a cross-sectional study of an important yet neglected problem in clinical practice

**DOI:** 10.3389/fnut.2023.1227840

**Published:** 2024-01-08

**Authors:** Behailu Terefe Tesfaye, Mengist Awoke Yizengaw, Tesema Etefa Birhanu, Dula Dessalegn Bosho

**Affiliations:** ^1^Department of Clinical Pharmacy, School of Pharmacy, Institute of Health, Jimma University, Jimma, Ethiopia; ^2^Human Anatomy Unit, Department of Biomedical Sciences, Institute of Health, Jimma University, Jimma, Ethiopia

**Keywords:** aged, inpatients, nutrition assessment, risk factors, Jimma

## Abstract

**Background:**

Malnutrition is a common geriatric syndrome affecting approximately half of the older population with a more pronounced occurrence rate in those hospitalized. It affects the physiology, and results in poor humanistic and clinical outcomes. In Africa, particularly in Ethiopia, albeit multiple studies are available on malnutrition in non-hospitalized older population, similar studies in inpatient settings are scarce. Therefore, this study was conducted with the intention to quantify the prevalence of malnutrition in older patients on inpatient admission and determine its associated factors.

**Methods:**

A total of 157 older inpatients aged 60 years and above were included in the present study. The data collection format was developed after an in-depth review of relevant literatures. The full Mini-Nutritional Assessment (MNA) tool was employed to assess the nutritional status on admission. Data completeness was checked thoroughly. Descriptive statistics and logistic regression analysis were conducted using STATA 15.0. The area under the receiver operating characteristic curve (ROC), Hosmer–Lemeshow test, and classification table were computed to evaluate the final model goodness-of-fit.

**Results:**

Of the total study subjects, 81% were malnourished (MNA score <17) and 17% were at risk for malnutrition (MNA score of 17.5–23.5). However, upon review of the patients’ medical charts, malnutrition diagnosis was recorded in only two patients. Rural residence (AOR = 2.823, 95%CI: 1.088, 7.324), self-reported financial dependence for expenses (AOR = 4.733, 95%CI: 1.011, 22.162), and partial dependence in functional autonomy on admission (AOR = 3.689, 95%CI: 1.190, 11.433) significantly increased the risk of malnutrition. The area under the ROC curve (0.754) and the Hosmer–Lemeshow test (*p* = 0.7564) indicated that the final model reasonably fits the data. The model`s sensitivity is 96.85%.

**Conclusion:**

In the present study, an alarmingly high prevalence of malnutrition was identified older inpatients. The problem went undiagnosed in a similar percentage of patients. Several available literatures indicate the presence of an association between nutritional status and patient outcomes, thus strict nutritional screening at inpatient admission and intervention are recommended with special emphasis for those from rural areas, with financial dependence, and with functional impairment on admission.

## Introduction

The global population dynamics show that the proportion of the older population is growing over time (1). Older populations are defined as those aged 65 years and above in developed countries, whereas for developing countries, such as Ethiopia, international organizations define older as a person aged 60 years and above (2). The proportion of individuals in this age group is projected to grow by two-fold, from 1 billion in 2020 to 2.1 billion in 2050. The projection also shows that in 2050, approximately 80% of older people will be residing in low- and middle-income countries (3). Ethiopia is also experiencing a demographic transition consistent with the global trend (4). This is presumed to affect almost all aspects of the global society, including the healthcare system (5).

Older people are usually fragile and vulnerable to malnutrition (6), which denotes failure to meet one's energy or protein requirements of high biological value (7). It is estimated that approximately half of the elderly population is affected by malnutrition (8), with a higher rate in hospitalized patients (9, 10). In this age group, low education, older age, anorexia of aging, multimorbidity, cognitive decline, polypharmacy, age-related functional decline, frailty, hospitalization, financial constraints, and empty nest syndrome are among the contributing factors to malnutrition (9, 11, 12). Furthermore, previously available studies mentioned factors such as sex (13, 14), age (13–15), rural residency (11), no formal education (11, 13, 14), low economic status (14, 15), functional impairment (11, 16, 17), smoking (18), having depression (11, 13–15), hospitalization history, alcohol consumption (14), and occupations (15) as malnutrition associated factors.

The occurrence of malnutrition results in deranged physiology and poor clinical outcomes. It affects the quality of life, increases the incidence of infection, causes sarcopenia, results in poor hospital outcomes, and increases the risk of life-threatening complications and mortality (9, 13, 15, 19, 20). As a result, malnutrition screen-and-treat policies are recommended in the hospital setting (21). However, malnutrition and nutritional aspects of some common clinical conditions of older age are often neglected in clinical practice (22). There are various nutritional assessment tools for screening malnutrition among hospitalized patients, but none of them are considered gold standard (23). The Mini-Nutritional Assessment (MNA) tool, a valid nutrition screening and assessment tool for elderly patients (24), has been employed in several nutritional assessment studies (25–27).

The prevalence of malnutrition among hospitalized older patients prominently varies across the available studies. In Europe, malnutrition has been reported in up to 80% of geriatric hospital patients (9), and at risk of malnutrition in nearly half of the elderly population (28). Studies from Australia recorded malnutrition and at risk for malnutrition in up to 88% of hospitalized elderly patients, respectively (25, 29). In one study from Brazil, moderate and severe malnutrition was recorded in more than half (55%) of the elderly patients (28), while in Asia, malnutrition prevalence was as high as 45% (26, 30–35) and at risk for malnutrition up to 67.1% (26, 30, 33–35) were reported.

In Africa, malnutrition among older people is a major challenge to the health care system and requires special pressing consideration. Almost all the available studies on the nutritional status of elderly people in the continent are from community settings (36–39). A review of observational studies from the community and pocket studies from the hospital settings in Africa revealed malnutrition prevalence of up to 56% in the hospitalized older population using the MNA tool (38). In other review studies involving mostly community-based and a few facility-based outpatient studies from Africa, malnutrition prevalence of 17% ranging from 1.8 to 39.47% was reported in the elderly population. The studies incorporated in the review assessed nutritional status using either body mass index (BMI) or MNA (36). In addition, a scoping review of community- and few facility-based, outpatient studies from sub-Saharan Africa indicated malnutrition prevalence between 6 and 54% in older people. The investigators of the studies included in the review employed BMI, the MNA tool, and Mid-Upper Arm Circumference (MUAC), alone or in combination, to assess nutritional status (37). On the other hand, a multi-country, multi-center prospective cohort study was conducted in selected hospitals from South Africa, Ghana, and Kenya. In the study, using Nutritional Risk Screening-2002^®^, 75.1% of patients screened at admission to the hospitals were at risk of malnutrition (40).

In previously available studies involving older patients, female sex, a low education level (41), older age (42, 43), unmarried (43, 44), polypharmacy, dysphagia, depression, low functional capacity, eating-related problems, lowered cognitive function (45), diabetes (26), alcohol abuse, and socio-economic status were described as independent risk factors of malnutrition (46–48).

In Ethiopia, a review of community-based studies that assessed undernutrition in the elderly population using BMI recorded a 20.53% pooled prevalence of undernutrition among the older population (39). Hitherto, studies on the prevalence of malnutrition among elderly inpatients in Africa, particularly in Ethiopia, are limited. Therefore, this study was conducted with the primary intention of determining the prevalence of malnutrition in older patients on admission to medical wards and determining its associated factors.

## Materials and methods

### Study design and participants

This cross-sectional study was conducted in the period from 6 September 2021 to 26 December 2022 in the medical wards of Jimma Medical Center (JMC), which is a Jimma University-affiliated institution in Jimma town, 352 km south-west of Addis Ababa. It is the only teaching and referral medical center in the southwestern part of the country with a bed capacity of 800, and provides services for a catchment population of about 15 million people. The center has over 1,000 health professionals, and 16 service departments, including emergency, ambulatory, internal medicine, pediatrics, adult and pediatric oncology, surgery, dentistry, physiotherapy, radiology, gynecology and obstetrics, sexual and reproductive health, and pharmacy (49, 50). In the present study, 157 consented older adult patients aged 60 years and above who were admitted to the medical wards (general unit, cardiac unit, stroke unit, and pulmonary unit) of JMC over the study period were considered. Participants with aphasia problems (*n* = 2) and re-admissions (*n* = 12) were excluded from the study.

### Sample size estimation and sampling procedure

Single population proportion formula was employed for calculating the sample size assuming a confidence level of 95%, a margin of error of 0.05, and a critical value (Z) of 1.96. For the purpose of this study, the prevalence of malnutrition in older people (*P* = 21.2%) was taken from a previous study in Ethiopia (14). In the year 2019–2020, approximately 398 older adult patients aged 60 years and above were admitted to the medical wards of JMC and this number was considered as a source population. The final minimum sample size was *n* = 157 after correction. Thus, 157 eligible older adult patients were consecutively assessed over the study period.

### Data collection tool and procedure

The data collection tool comprised both, standard tools and tools designed after an in-depth review and extraction from relevant literatures. It was designed to capture relevant socio-demographic, behavioral, functional, clinical, nutritional, and related information of the participants. The tool was translated to two locally predominant languages (Afan Oromo and Amharic) and back translated into English to check the consistency. Two data collectors (Bachelor's degree nurses) were trained on the data collection tool and procedure. Prior to the actual data collection period, a pre-test was conducted. The data collection procedure throughout the study period was carried out under close supervision of the investigators. Patient charts, laboratory results, patient/caregiver interviews, and practitioners in charge were the sources of data for the present study. All data were collected from eligible patients within 48 h of admission to the ward. Each patient’s diagnosis was reviewed from their medical chart to check on how many of these patients were diagnosed with malnutrition over their hospital stay.

### Study variables

The independent variables were socio-demographic and related variables [age, sex, residence, marital status, educational level, patient’s current working status, occupation, and self-reported financial dependence (not able to cover their personal expense)]; behavioral, functional, and related information [alcohol drinking, cigarette smoking, and khat chewing history; recent traditional medicine use history, cohabitation, and activities of daily living (ADL)]; clinical and related information (presence of past medical history, hospitalization in the previous 1-year, psychological condition on admission). Katz Index of independence was employed to assess ADL, which indicates the functional health status of older patients on admission (51). The tool assesses performance in six daily living functions (eating, dressing, bathing, transferring, continence, and toileting), each of which is assigned a score of 1 or 0. Accordingly, patients are categorized as independent (full function), partially dependent (moderate impairment), or dependent (severe functional impairment) if they scored 6, 3–5, and 2 or less points, respectively. The shortened self-report form of the Geriatric Depression Scale (GDS) which comprised 15 items, was used to objectively assess the psychological condition of older patients on admission (52). Each question in GDS has two alternative responses, either yes or no, and the patients are categorized as having no psychological problem (0–4), mild depression/dementia (5–9), or severe depression/dementia (10–15). The outcome of this study is the nutritional status of older patients on admission to medical wards based on the full MNA tool score.

### Nutritional assessment

The nutritional assessment on admission was carried out using the full MNA tool. The MNA tool (24, 53) is recommended as a screening tool for malnutrition in older inpatients. The tool has been evaluated in the older population in Ethiopia (54). It is widely employed in studies and has good reliability and validity. It has 18 questions that can be categorized into four parts: anthropometric, overall assessment, diet assessment, and subjective assessment. The summative score is 30 points: 24–30 points indicate good nutrition; 17.0–23.5 points indicate risk of malnutrition; and less than 17.0 points indicate malnutrition. MNA has 96% sensitivity and 98% specificity (55). In assessing nutritional status on admission, body mass index (BMI) was calculated using the standard formula: BMI = weight in kg/(height in m)^2^. The weight of the study participants was measured using beam balance. For taking the weight of the participant, each of them was requested to take off their shoes and heavy clothes, including jackets, jerseys, and belts; the balance was calibrated; and the figure was approximated to the nearest 0.01 kg. For measuring height, a seca vertical height scale was used. Patients were asked to take off their shoes and stand upright in the middle of the board. Thereafter, height was measured to the nearest 0.01 cm by making the participant’s back of the head, the shoulder blades, the buttocks, and the heels to touch the measuring board. Upon encountering patients who cannot erect upright because of their illness, their height was estimated from demi-span (56). In line with a standard recommendation, the length from the sternal notch to the finger roots (demi-span) was measured by a flexible validated plastic tape with the patient sitting upright or recumbent depending on the patient’s abilities. Then, height was estimated as follows: for female individuals: Height in cm = (1.35 × demi-span in cm) + 60.1; for male individuals: Height in cm = (1.40 × demi-span in cm) + 57.8.

### Data management and statistical analysis

Data were checked for completeness and accuracy during the data collection period, entry, and before the analysis. Epi data version 4.2.0.0 and STATA V.15.0 were employed for data entry and analysis, respectively. Categorical variables were described in frequency and percentage. Upon performing the Shapiro-Wilk test, the distribution of continuous variables was non-normal, thus presented in the median and interquartile range (IQR). For the binary logistic regression model, nutritional status was dichotomized into 0 = no malnutrition (normal and at risk of malnutrition) and 1 = malnutrition. Candidate variables for regression analysis were selected based on the existing literatures, and all variables with a *p* < 0.25 in simple logistic regression were incorporated into multiple logistic regression analysis. Collinearity was checked using the variance inflation factor (VIF); all factors included in the multiple logistic regression analysis had a VIF of <10 (maximum 1.48). The interaction was evaluated using statistical models by including product terms for two independent variables presumed to have interactions in the model. The goodness-of-fit of the final model was evaluated by computing the area under the receiver operating characteristic curve (ROC), Hosmer–Lemeshow test, and classification table. In all the analyses, a two-tailed *p* < 0.05 was used to declare statistical significance.

## Results

The median age of the participants was 65 years, and most of them were men (82.2%). Of the total study subjects, farmers accounted for 24.2%, and self-reported financial independence for healthcare expenditure was captured in over three-fourths (78.3%). The socio-demographic and behavioral information of the study subjects are shown in Table 1.

**Table 1:** Socio-demographic and behavioral information of the study subjects.

Variables	n (%)
Sex	
Male	129 (82.2)
Female	28 (17.8)
Age, Median (IQ)	65 (60–70)*
Young old (60–74)	121 (77.8)
Middle old (75–84)	30 (18.5)
Older old (≥85)	6 (3.7)
Residence	
Urban	32 (20.4)
Rural	125 (79.6)
Marital status	
Never married (single)	1 (0.6)
Married	129 (82.2)
Divorced	8 (5.1)
Widowed	19 (12.1)
Educational status	
No formal education	148 (94.3)
Primary education (grade 1–8)	6 (3.8)
Collage and above	3 (1.9)
Occupation	
No job	65 (41.4)
Farmer	38 (24.2)
Housewife	23 (14.7)
Retired	20 (12.7)
Merchant	6 (3.8)
Daily laborer	4 (2.6)
Public employee	1 (0.6)
Currently working	
Yes	49 (31.2)
No	108 (68.8)
Self-reported financial dependence	
Dependent	34 (21.7)
Independent	123 (78.3)
Alcohol consumption history	
Yes	45 (28.7)
No	112 (71.3)
Cigarette smoking history	
Yes	38 (24.2)
No	119 (75.8)
Khat chewing history	
Yes	113 (72)
No	44 (28)
Cohabitation	
With spouse and children	80 (51)
With spouse	41 (26.1)
With children	29 (18.5)
Alone	7 (4.5)

*Median (IQR), IQR, interquartile range.

### Clinical and related information of the study participants

On assessing the activities of daily living using the Katz score, 78% of the participants had impairment in their functional autonomy at admission. Past medical history was captured in 65.6% of the participants, and each of these patients had three known diseases on average. A little above one-third (33.8%) of the participants had a previous hospitalization history. The functional, clinical, and related information of the study subjects is presented in Table 2.

**Table 2:** Functional, clinical, and related information of the study subjects.

Variables	n (%)
Katz score, median (IQR)	3 (0–6)*
Functional autonomy	
Dependent (2 or less)	64 (40.8)
Partially dependent (3–5)	49 (31.2)
Fully independent (6)	44 (28)
Past medical history	
Yes	103 (65.6)
No	54 (34.4)
Number of diseases diagnosed on admission, median (IQR)	3 (3–4)*
Top five previously diagnosed diseases	
Heart failure	25 (15.9)
Hypertension	49 (31.2)
Type 2 diabetes mellitus	13 (8.3)
Bronchial asthma	12 (7.6)
Ischemic heart disease	8 (5.1)
Previous hospitalization history	
Yes	53 (33.8)
No	104 (66.2)
Surgical procedure history	
Yes	8 (5.1)
No	149 (94.9)

*Median (IQR), IQR, interquartile range.

### Nutritional status of the study participants

**Figure 1:**
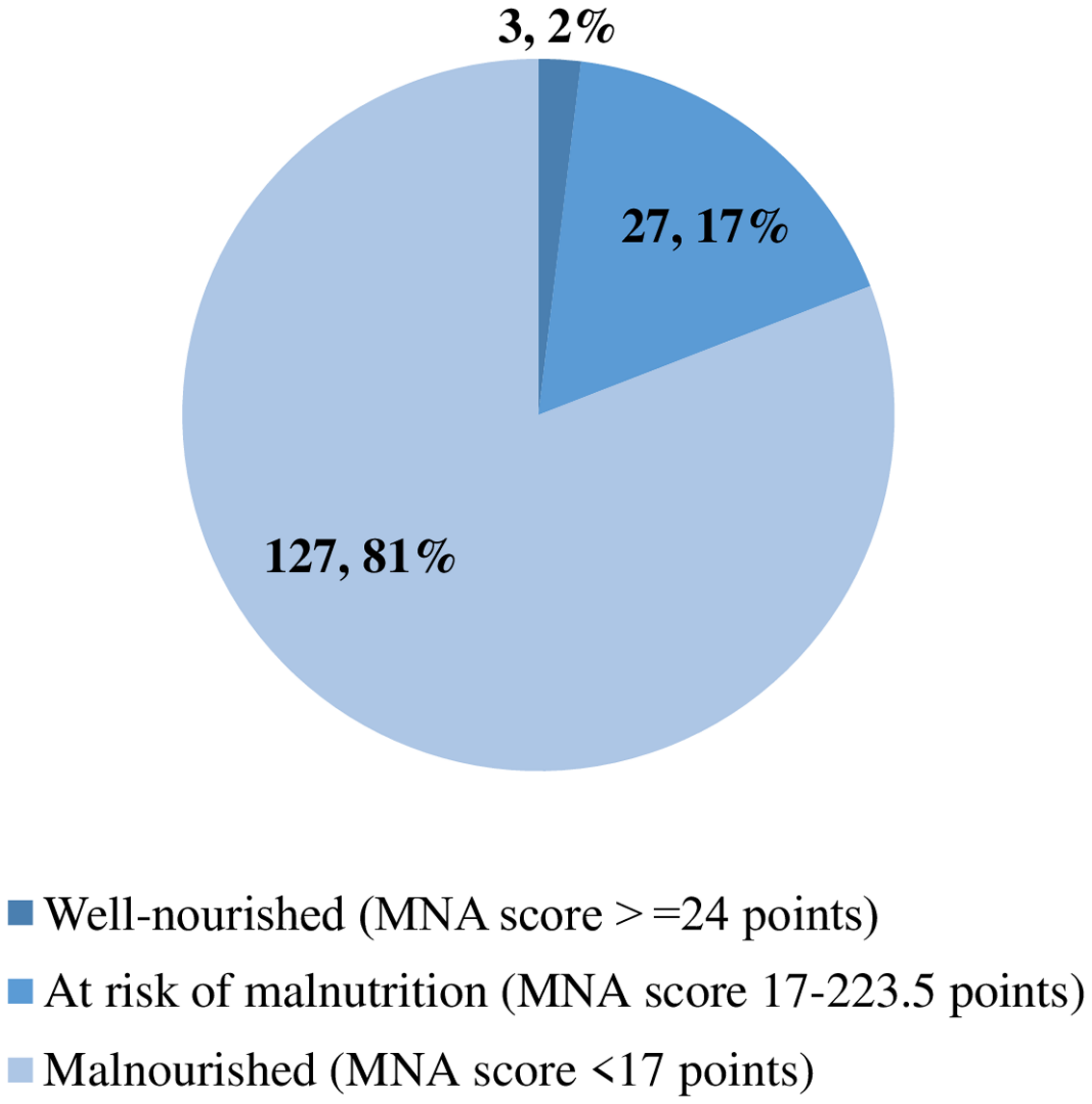
The proportion of hospitalized elderly patients according to their nutritional category.

On the MNA tool-based nutritional status assessment at admission, 81% of the study participants had a score less than 17 and were categorized as malnourished. However, upon a thorough review of patients` medical charts over the hospital stay, malnutrition diagnosis was recorded in only two. The prevalence of elderly inpatients’ nutritional category is depicted in Figure 1.

The full MNA tool questions with data captured from the study subjects are presented in Table 3.

**Table 3:** Full MNA tool questions with data captured from the study subjects.

Variables	n (%)
Has food intake declined over the past 3 months	
Severe decrease in food intake	61 (38.8)
Moderate decrease in food intake	75 (47.8)
No decrease in food intake	21 (13.4)
Weight loss during the last 3 months	
Weight loss greater than 3 kg	23 (14.6)
Dose not know	99 (63.1)
Weight loss between 1 and 3 kg	19 (12.1)
No weight loss	16 (10.2)
Mobility	
Bed or chair bound	79 (50.3)
Able to get out of bed/chair but does not go out	66 (42.0)
Goes out	12 (7.7)
Has suffered psychological or acute disease in the past 3 months	
Yes	19 (12.1)
No	138 (87.9)
Neuropsychological problem	
Severe dementia or depression	32 (20.4)
Mild dementia	92 (58.6)
No psychological problem	33 (21.0)
Body mass Index (BMI)	
BMI less than 19	57 (36.3)
BMI 19 to less than 21	67 (42.7)
BMI 21 to less than 23	19 (12.1)
BMI 23 or greater	14 (8.9)
Lives independently (not in a nursing home or hospital)	
Yes	4 (2.6)
No	153 (97.4)
Takes more than 3 prescription drugs per day	
Yes	143 (91.1)
No	14 (8.9)
Pressure sores or skin ulcers	
Yes	21 (13.4)
No	136 (86.6)
How many full meals does the client eat daily?	
1 meal	11 (7.0)
2 meal	82 (52.2)
3 meal	64 (40.8)
Meat, fish, or poultry every day	
If 0 or 1 yes	69 (44)
If 2 yes	77 (49.0)
If 3 yes	11 (7)
Consume two or more servings of fruit or vegetables per day	
No	119 (75.8)
Yes	38 (24.2)
How much fluid (water, juice, coffee, tea, milk…) is consumed per day?	
Less than 3 cups	53 (33.8)
3–5 cups	95 (60.5)
More than 5 cups	9 (5.7)
Mode of feeding	
Unable to eat without assistance	54 (34.4)
Self-fed with some difficulty	54 (34.4)
Self-fed without any problem	49 (31.2)
Self-view of nutritional status	
View self as being malnourished	41 (26.1)
Is uncertain of nutritional status	89 (56.7)
View self as having no nutritional problem	27 (17.2)
In comparison with other people of the same age, how does the client consider his /her health status	
Not as good	5 (3.2)
Dose not know	132 (84.1)
As good	15 (9.5)
Better	5 (3.2)
Mid–arm circumference (MAC) in cc	
MAC less than 21	93 (59.2)
MAC 21–22	31 (19.8)
MAC greater than 22	33 (21.0)
Calf circumference (CC) in cm	
CC less than 31	132 (84.1)
CC 31 or greater	25 (15.9)

### Factors associated with the nutritional status of elderly patients

Six variables achieved a *p* < 0.25 in the simple logistic regression analysis; only residence (*p* = 0.017) and financial dependence (*p* = 0.041) were significantly associated with nutritional status. Subsequently, multiple logistic regression analysis was performed by incorporating the main variables achieving a *p* < 0.25 and some pertinent interaction terms (financial dependence#residence, financial dependence#sex, and functional autonomy#sex). However, none of the interaction terms were statistically significant, thus removed from the model and the analysis was rerun without the interaction terms. In the final model, significantly higher odds of malnutrition were observed in older patients from rural areas (AOR = 2.823, 95%CI: 1.088, 7.324, 0.033), with financial dependence (AOR = 4.733, 95%CI: 1.011, 22.162, 0.048), and with partial dependence in functional autonomy on inpatient admission (AOR = 3.689, 95%CI: 1.190, 11.433, 0.024). The model achieved an area under the ROC curve of 0.754 and an insignificant Hosmer–Lemeshow test *p*-value (0.756) indicating a good fit. On classification table analysis, the model correctly classified 82.20% of the participants achieving a sensitivity of 96.85%. The regression analysis is shown in Table 4.

**Table 4:** Logistic regression analysis to identify factors associated with the nutritional status of elderly patients on hospital admission.

Variables	COR (95%CI)	*P*-value	AOR (95%CI)	*P*-value
Sex				
Male	1			
Female	2.206 (0.619, 7.858)	0.222	1.347(0.343, 5.295)	0.670
Age				
60–74	1			
≥75	1.615 (0.569, 4.577)	0.368		
Residence				
Urban	1			
Rural	2.922 (1.215, 7.030)	0.017	2.823 (1.088, 7.324)	0.033
Marital status				
Single/divorced/widowed	1.515 (0.483, 4.748)	0.476		
Married	1			
Educational status				
No formal education	1			
Formal education	2.241 (0.527, 9.527)	0.275		
Financial dependence				
Independent	1			
Dependent	4.716(1.063, 20.913)	0.041	4.733 (1.011, 22.162)	0.048
Alcohol consumption history				
Yes	1.1306(0.462, 2.767)	0.788		
No	1			
Cigarette smoking history				
Yes	0.690 (0.285, 1.671)	0.412		
No	1			
Khat chewing history				
Yes	1.368 (0.581, 3.215)	0.473		
No	1			
Traditional medicine use				
Yes	1.005 (0.312, 3.237)	0.994		
No	1			
Autonomy for ADL		0.056		
Fully independent	1			
Partially dependent	3.344 (1.154, 9.691)	0.026	3.689 (1.190, 11.433)	0.024
Dependent	2.52 (0.998, 6.362)	0.050	2.461 (0.914, 6.623)	0.075
Past medical history				
Yes	0.943 (0.406, 2.190)	0.892		
No				
Previous hospitalization history				
Yes	1.237 (0.522, 2.929)	0.629		
No	1			
Surgical procedure history				
Yes	0.694 (0.133, 3.623)	0.665		
No	1			
Number of diseases diagnosed on admission	1.150 (0.882, 1.498)	0.301		
Bronchial asthma				
Yes	0.437 (0.122, 1.561)	0.202	0.476 (0.118, 1.921)	0.297
No	1			
Heart failure				
Yes	1.886 (0.525, 6.770)	0.331		
No	1			
Hypertension				
Yes	0.617 (0.270, 1.407)	0.251		
No	1			
Ischemic heart disease				
Yes	0.694 (0.133, 3.622)	0.665		
No	1			
Type 2 diabetes mellitus				
Yes	0.336 (0.101, 1.113)	0.074	0.374 (0.096, 1.261)	0.108
No	1			

ADL, activity of daily living; AOR, adjusted odd ratio; COR, crude odds ratio.

## Discussion

This single-center prospective cross-sectional study is a pioneer in quantifying the prevalence of malnutrition and determining associated factors in older patients on admission to medical wards in Ethiopia. Patients were assessed for nutritional status within 48 h of admission to medical wards using the full MNA tool. Accordingly, almost all admitted elderly patients (98%) were either malnourished (81%) or at risk of malnutrition (17%). However, clinicians recorded malnutrition diagnosis in only two patients’ medical charts, which indicates that malnutrition diagnosis is neglected in almost all patients.

In the previous studies from Ethiopia, the pooled prevalence of undernutrition among the older population was 20.53% (39); however, the included studies were entirely community based. In Egypt, a similar study involving 194 older patients admitted with cancer reported malnutrition in 33% of the participants (57). In the study from Egypt (57), the study participants were a cohort of patients with cancer, which along with other dissimilarities in the study participants’ characteristics might have contributed to a lower prevalence of malnutrition as compared to our result. In Europe, malnutrition has been reported in as high as 80% of older patients (9), which is consistent with our findings. Other similar studies from Europe (27, 28), Australia (25, 29), Brazil (28), and Asia (26, 30–35) reported malnutrition far lower than our findings. This could be attributed to potential differences in the study participants’ characteristics. The observed, alarmingly high prevalence of malnourished patients in our study requires immediate intervention. In fact, the elderly often become dependent on others for their food and nutrition (58), in the context of poor social support status seen in Ethiopia (59), huge vulnerability to malnutrition is expected. The policy direction for old age and food insecurity seen in Ethiopia due to underlying multifactorial causes might also contribute to the observed malnutrition prevalence in older inpatients (60). Evidence also shows that medical comorbidity, physical impairments (9, 10), and hospitalization (9, 11) affect nutritional status in elderly people. In the present study, a higher proportion of elderly patients had a past medical history (65.6%) and physical impairment (59.2%). However, in this study, the presence of past medical history was not significantly associated with malnutrition.

In this study, significantly higher odds of malnutrition were observed in older patients who were from rural areas. This finding is in line with other study findings from Ethiopia (18, 61). The possible difference in socio-economic status and dietary habits may have increased rural residents' risk for malnutrition. Also, several studies involving elderly people reported the existence of a significant association between functional autonomy and malnutrition (62, 63). A similar finding has also been observed in our study where the risk of malnutrition is high among patients who do have partial impairment in performing activities of daily living on admission in contrast to those who do not. Older patients with partial dependence on functional autonomy on admission had over 3.6-fold increased risk for malnutrition as compared to those who are fully independent. This is due to the fact that these patients lack functional autonomy to care after oneself and to prepare and eat foods requiring the attention of others (64). This fact is corroborated by our study finding in which most of the participants (86.6%) believed their food intake was decreased either moderately or severely.

Food and nutrition insecurity due to lower economic status also increases the risk for malnutrition (48), which is also observed in our study where older patients who were financially dependent for their expenses had a 4.73-fold increased risk for malnutrition as compared to their counterparts. This finding is consistent with other similar studies (15, 48). On the other hand, previously existing studies described female sex, low education level (41), older age (42, 43), unmarried (43, 44), polypharmacy, dysphagia, neuropsychological problem (45), alcohol abuse, and tobacco use as independent risk factors of malnutrition. The relatively longer life expectancies than men and the higher likelihood to encounter adverse economic and social circumstances in old age are assumed to increase the risk of malnutrition in women. As people get older, age-related alterations, such as physical frailty, loss of taste and poor appetite, the feeling of worthlessness, and a sense of neglect, can impact eating habits with adverse consequences on nutritional status (65). Beyond their impact on nutritional status, educational level, marital status, and sex, they were also found to affect discharge outcomes (66). In the present study, as seen in Table 4, the crude odds ratio shows an increased risk of malnutrition in the female sex, in patients with no formal education, older age, marital status of single, and alcohol abuse, however, none of them achieved a statistically significant association with malnutrition. This could be due to the small sample size employed in our study. Regarding polypharmacy (prescription drugs taken by the patients) and neuropsychological problems, these variables are already components of the full MNA tool used for outcome measurement (see Table 3), thus rationally excluded from considering them as independent variables in the logistic regression analysis in the present study.

From the results of the present study and the potential complications of undernutrition in elderly patients, the investigators recommend the installation of a system for the prevention, early detection, and management of malnutrition in elderly patients. First, for the prevention of malnutrition and its complications, Ethiopia: Food-Based Dietary Guidelines (67) encourage elderly people to take adequate fluid and diversified diets, such as meat, poultry products, fruits, and vegetables to prevent malnutrition. However, practically, given that the elderly are one of the poorest and most marginalized sections of the population in Ethiopia (68, 69), the authors have reservation on the possibility for successful implementation of governmental and non-governmental stakeholders. Second, the authors of the present study recommend early screening of elderly patients for nutritional status and managing those affected by malnutrition with oral nutritional supplementation, such as plumpy nut, and diet enhancement in the hospital settings according to the national and relevant international guidelines, such as European Society for Clinical Nutrition and Metabolism (70). In doing so, special consideration for older patients with functional impairment on admission might be helpful. Finally, we also recommend a multicenter, large sample size study to increase the power of generalizability of the findings in the present study.

In this study, the collection of relevant data objectively from the older patients using standardized techniques and the exploratory nature of the study for the setting, as well as for Ethiopia, could be mentioned as merits. However, the study has some limitations. The single-center consideration and the small sample size employed may affect the accuracy and generalizability of the findings. Also, the causal association cannot be assessed due to the intrinsic nature of the cross-sectional design. Some pertinent variables, such as a number of family size, the presence of eating-related problems, and biochemical parameters for nutritional assessments were not addressed in the present study. We could not also rule out the possibility of response bias where some respondents may not actually answer some questions truthfully, which may distort study results.

In conclusion, using the full MNA tool, an alarmingly high prevalence of malnutrition and at risk of malnutrition was identified. Nevertheless, the problem went undiagnosed in a similar proportion of the patients. Owing to the existence of multiple evidences on the adverse clinical and humanistic consequences of malnutrition, the authors recommend designing immediate strategies for routine screening at admission and nutritional intervention with special emphasis on older patients from rural areas, with financial constraints, and with functional impairment on admission. Further large sample size study, incorporating pertinent variables missed in the present study and possibly assessing the clinical consequences of malnutrition in older patients in the local context is recommended.

## Data availability statement

The original contributions presented in the study are included in the article/supplementary material, further inquiries can be directed to the corresponding author.

## Ethics statement

The study involving humans was approved by the Institutional Review Board (IRB) of Jimma University. The study was conducted in accordance with the local legislation and institutional requirements. The participants provided their written informed consent to participate in this study.

## Author contributions

BT, MY, and DB substantially contributed to the data acquisition and results explanation and drafted the manuscript. BT performed the data analysis. TB contributed to the explanation of the results and revised the manuscript. All authors have read and agreed to the published version of the manuscript.
